# Promotion of Germination Using Hydroxamic Acid Inhibitors of 9-*cis*-Epoxycarotenoid Dioxygenase

**DOI:** 10.3389/fpls.2017.00357

**Published:** 2017-03-20

**Authors:** Sajjad Z. Awan, Jake O. Chandler, Peter J. Harrison, Martin J. Sergeant, Timothy D. H. Bugg, Andrew J. Thompson

**Affiliations:** ^1^School of Life Sciences, University of WarwickCoventry, UK; ^2^Cranfield Soil and Agrifood Institute, Cranfield UniversityCranfield, UK; ^3^Department of Chemistry, University of WarwickCoventry, UK

**Keywords:** seed germination, abscisic acid, hydroxamic acid, 9-*cis*-epoxycarotenoid dioxygenase, dormancy, chemical genetics

## Abstract

Abscisic acid (ABA) inhibits seed germination and the regulation of ABA biosynthesis has a role in maintenance of seed dormancy. The key rate-limiting step in ABA biosynthesis is catalyzed by 9-*cis*-epoxycarotenoid dioxygenase (NCED). Two hydroxamic acid inhibitors of carotenoid cleavage dioxygenase (CCD), D4 and D7, previously found to inhibit CCD and NCED *in vitro*, are shown to have the novel property of decreasing mean germination time of tomato (*Solanum lycopersicum* L.) seeds constitutively overexpressing *LeNCED1*. Post-germination, D4 exhibited no negative effects on tomato seedling growth in terms of height, dry weight, and fresh weight. Tobacco (*Nicotiana tabacum* L.) seeds containing a tetracycline-inducible *LeNCED1* transgene were used to show that germination could be negatively and positively controlled through the chemical induction of gene expression and the chemical inhibition of the NCED protein: application of tetracycline increased mean germination time and delayed hypocotyl emergence in a similar manner to that observed when exogenous ABA was applied and this was reversed by D4 when *NCED* expression was induced at intermediate levels. D4 also improved germination in lettuce (*Lactuca sativa* L.) seeds under thermoinhibitory temperatures and in tomato seeds imbibed in high osmolarity solutions of polyethylene glycol. D4 reduced ABA and dihydrophaseic acid accumulation in tomato seeds overexpressing *LeNCED1* and reduced ABA accumulation in wild type tomato seeds imbibed on polyethylene glycol. The evidence supports a mode of action of D4 through NCED inhibition, and this molecule provides a lead compound for the design of NCED inhibitors with greater specificity and potency.

## Introduction

Seed dormancy is “an innate seed property that defines the environmental conditions in which the seed is able to germinate” (Finch-Savage and Leubner-Metzger, 2006). Therefore, dormant seeds may not germinate even in conditions otherwise suitable for germination. Dormancy mechanisms allow seeds to germinate at a time and location where subsequent conditions will allow plant reproduction. The mechanisms vary between plant species, but physiological seed dormancy is the most prevalent dormancy class (Baskin and Baskin, 2004) and is the focus of this study. During seed maturation, primary dormancy is induced, preventing vivipary, and can be sustained beyond seed maturation. Primary dormancy can be released in dry seeds through after-ripening, or in imbibed seeds by particular environmental stimuli such as short cold or warm periods and this reduction in dormancy depth enables seeds to germinate over a wider range of environmental conditions. Dormancy depth can also increase in response to prolonged absence of conditions which allow germination, and this is known as secondary dormancy (Finch-Savage and Leubner-Metzger, 2006). The mechanisms that control dormancy are complex and vary between species. In brief, environmental factors are perceived by the seed, and then signaling mechanisms modulate embryo growth potential and the strength of constraints imposed by the maternal tissues. Germination occurs when the embryo growth potential is sufficient to rupture the seed coat and allow radicle emergence. The balance of the levels of, and sensitivity to, the antagonistic phytohormones abscisic acid (ABA), and gibberellic acid (GA) is a predominant regulator of seed dormancy and germination (Kucera et al., 2005; Finkelstein et al., 2008).

Depending on the seed, the outer testa and/or the inner endosperm constrain the developing embryo and contribute to dormancy. ABA delays endosperm rupture in a variety of species including tobacco (Leubner-Metzger, 2003), coffee (da Silva et al., 2004), Arabidopsis and cress (Müller et al., 2006). ABA exerts its action, in part, by inhibiting induction of β-1,3-glucanase, an enzyme that weakens the endosperm in tobacco (Leubner-Metzger and Meins, 2000) and other Solanaceous seeds (Petruzzelli et al., 2003). ABA may also play a role in controlling testa rupture in tomato, since ABA-deficient mutants exhibit a thinner testa (Hilhorst and Downie, 1996). As well as affecting the seed coat, ABA can inhibit embryo growth potential as shown in coffee (da Silva et al., 2004), barley (Wang et al., 1995), and sunflower (Lepagedegivry and Garello, 1992), for example.

It is known that ABA derived from the developing embryo has a critical role in initiating dormancy because ABA biosynthetic mutants of many species including Arabidopsis (Koornneef et al., 1982; Lefebvre et al., 2006), tomato (Groot and Karssen, 1992), *Nicotiana plumbaginifolia* (Marin et al., 1996), and maize (Tan et al., 1997) form non-dormant and viviparous seeds. Whether ABA produced at this stage maintains dormancy is open to debate. In some studies, the breaking of dormancy by over-ripening is associated with a decline in seed ABA content. This has been demonstrated in barley (Benech-Arnold et al., 1999b), *N. plumbaginifolia* (Grappin et al., 2000) and pine (Feurtado et al., 2004) and to a lesser extent in Arabidopsis (Ali-Rachedi et al., 2004). However, seeds of Arabidopsis mutants unable to synthesize or perceive ABA are still able to undergo the after-ripening process, which results in distinguishable changes in the transcriptome (Carrera et al., 2008). Furthermore, dormancy does not always correlate with dry seed ABA concentration (Millar et al., 2006).

Hence dormancy does not seem to be maintained in dry seed by high residual levels of ABA produced during seed maturation, but rather by *de novo* ABA synthesis upon imbibition. *De novo* ABA biosynthesis occurs in dormant, but not non-dormant seeds of *N. plumbaginifolia* (Grappin et al., 2000), Arabidopsis (Ali-Rachedi et al., 2004), and barley (Benech-Arnold et al., 2006). Fluridone and norflurazon, which inhibit phytoene desaturase, have been used in a number of studies to inhibit *de novo* ABA biosynthesis: these inhibitors, when present in imbibed dormant seeds of Arabidopsis (Ali-Rachedi et al., 2004), barley (Leymarie et al., 2009), potato (Alvarado and Bradford, 2005), and tobacco (Grappin et al., 2000), cause an increased or hastened germination. The *de novo* synthesis mechanism appears to be highly conserved because imbibing seed with fluridone can break dormancy in a wide range of seeds from the parasitic plants *Orobranche* and *Striga* (Kusumoto et al., 2006; Song et al., 2006) to trees such as cedar and pine (Schmitz et al., 2001; Feurtado et al., 2007). Transgenic evidence also supports a role for ABA biosynthesis during imbibition in seed dormancy maintenance: constitutive overexpression of *LeNCED1* in tomato reduced or completely blocked germination in different transgenic lines, an effect that was completely reversed if norflurazon was applied during imbibition (Thompson et al., 2000); induction of a dexamethasone inducible *NCED* gene in *Nicotiana plumbaginifolia* delayed germination by about 4 days and reduced final germination to about 60% (Qin and Zeevaart, 2002) and methoxyfenozide-inducible overexpression of *AtNCED6* enhanced dormancy in imbibed seeds of Arabidopsis (Martínez-Andújar et al., 2011). Increased ABA biosynthesis and sensitivity is also associated with dormancy cycling in Arabidopsis (Footitt et al., 2011), high-temperature inhibition of germination of Arabidopsis (Toh et al., 2008), lettuce (Argyris et al., 2008), and barley (Leymarie et al., 2009), and the regulation of Arabidopsis seed germination by phytochrome (Seo et al., 2006). Thus, synthesis of ABA in imbibed seed appears to be critical to preventing germination in dormant seed.

The herbicides fluridone and norflurazon inhibit phytoene desaturase, and block ABA biosynthesis through depletion of carotenoid precursors. Despite extensive use in germination studies, these inhibitors are not ideal because carotenoids play vital functions in seedling growth and development, and a reduction in carotenoid synthesis leads to chlorophyll bleaching and seedling death. Other carotenoid-derived small molecules such as the strigolactones (SLs; Umehara et al., 2008), the BYPASS1 signal (Kang et al., 2008; Adhikari et al., 2013), and a lateral-root branching signal (Van Norman et al., 2014) could also be affected by the action of these herbicides thereby making the interpretation of their effects complex. Indeed, fluridone increased germination in annual ryegrass (Goggin et al., 2009), Orobanche (Chae et al., 2004), and Sorghum seeds (Benech-Arnold et al., 1999a) without decreasing endogenous ABA levels, suggesting the possibility that fluridone was acting through another mechanism, perhaps by reducing the production of other carotenoid-derived hormones that are involved in dormancy. A recent study has highlighted a role for SLs in the alleviation of germination thermoinhibition in Arabidopsis (Toh et al., 2012). Thus, in order to specifically study the role of endogenous ABA in dormancy, a more targeted ABA synthesis inhibitor is required that will also allow studies to be extended into seedling establishment. The cleavage of 9-*cis*-epoxycarotenoids to xanthoxin by NCED is the first committed and rate-limiting step in ABA biosynthesis; it therefore represents an ideal target for selective inhibition of ABA biosynthesis.

To date, NCED inhibitor compounds are relatively inefficient at inducing germination. The first known inhibitor of the conversion of 9-*cis*-violaxanthin and 9′-*cis*-neoxanthin to xanthoxin was nordihydroguaiaretic acid (NDGA), a lipoxygenase inhibitor, which was later shown also to inhibit NCED but has little effect on seed germination (Creelman et al., 1992; Han et al., 2004). Abamine was developed as a more specific inhibitor of NCED which increased radicle length in cress seeds after 24 h imbibition (Han et al., 2004) and increased rate of seed germination in Arabidopsis C24 (Han et al., 2004; Li et al., 2012), but abamine strongly inhibits Arabidopsis seedling growth at 100 μM due to non-specific effects (Kitahata et al., 2006). A derivative of abamine, abamineSG is a more potent inhibitor of NCED and does not inhibit growth of Arabidopsis seedlings (Kitahata et al., 2006), but it has not been reported if abamineSG improves seed germination. Further, a set of sesquiterpene-like compounds, which inhibit NCED and ABA synthesis in plants, actually decreased seed germination rate in Arabidopsis (Boyd et al., 2009).

In another study, hydroxamic acid inhibitors were developed (Sergeant et al., 2009) that specifically inhibit CCDs that cleave carotenoids at different positions in the tetraterpenoid chain. NCED, a type of CCD, cleaves the 9-*cis*-epoxycarotenoid ABA precursors at the 11–12 and 11′–12′ positions (Schwartz et al., 1997), whereas, for example, LeCCD1 cleaves carotenoids at the 9–10 and 9′–10′ positions (Simkin et al., 2004). Specificity was partially achieved by varying the distance between an iron-chelating hydroxamic acid moiety and an aromatic ring mimicking the cyclic terminus found in some carotenoids (Sergeant et al., 2009). Some of the compounds (Table 1) show useful degrees of *in vitro* NCED inhibition and thus represent candidates for *in vivo* ABA biosynthesis inhibitors. In this paper, we investigate the effects of the hydroxamic acid inhibitors on seed germination in several species and scenarios where NCED expression is increased either transgenically (in both constitutive and inducible systems), or physiologically.

**Table 1:** **Structure of carotenoid cleavage dioxygenase inhibitors and *in vitro* inhibition efficacy against *LeCCD1a* and *LeNCED1***.

**Inhibitor**				**Inhibition @ 100 μM (%)**		**Structure**
**Class**	**Name**	**X**	**Y**	***LeCCD1a* (9,10 / 9′,10′)**	***LeNCED1* (11′,12′)**	
Abamine		–	–	35 ± 15^a^	20	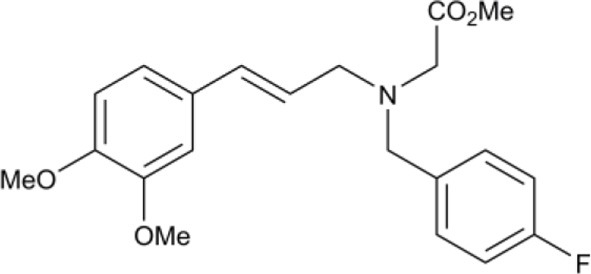
Aryl-C_2_N	D1	4-OH	H	>95	27	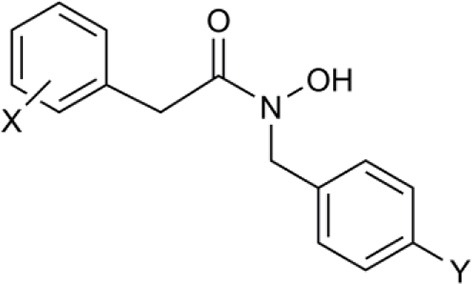
	D2	4-OH	F	>95	29	
	D3	3,4-(OH)_2_	F	>95	4	
	D4	4-OMe	F	>95	33	
	D5	3,4-(OMe)_2_	H	>95	8	
	D6	3,4-(OMe)_2_	F	>95	18	
	D7	3,4-OCH_2_O	F	>95	33	
Aryl-C_3_N (class 1)	D8	3,4-(OMe)_2_	H	61	40	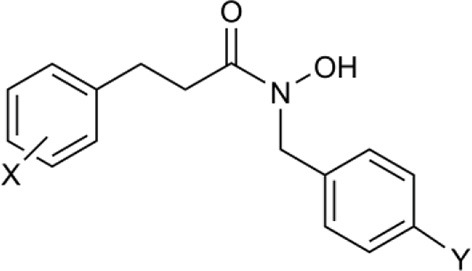
	D14	3,4-(OMe)_2_	F	nd	nd	
	D15	4-OMe	F	70^a^	24^a^	
Aryl-C_3_N (class 2)	D12	–	–	26	11	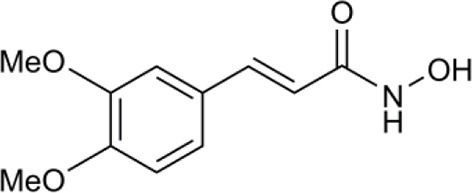
	B2	–	–	47^b^	13^b^	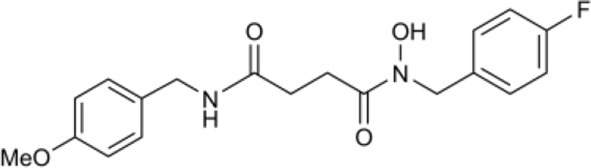

a *Van Norman et al. (2014)*;

b *Unpublished data. All other data from Sergeant et al. (2009). nd, not determined*.

## Materials and methods

### Hydroxamic acid inhibitors

Synthesis of all inhibitors was previously reported (Sergeant et al., 2009; Van Norman et al., 2014), with the exception of B2 and D14. Compound D14 was synthesized by previously reported methods (Sergeant et al., 2009), using 4-fluorobenzyl bromide and 3,4-dimethoxycinnamic acid as precursors. Compound B2 was also synthesized via previously reported coupling methods, using 4-fluorobenzyl bromide and N-succinyl-(4-methoxybenzylamine) as precursors. N-succinyl-(4-methoxybenzylamine) was synthesized via reaction of 4-methoxybenzylamine with succinic anhydride in tetrahydrofuran. Spectroscopic data for B2 and D14 as novel compounds are given here: B2: *N*-(4-fluorobenzyl)-(4-methoxybenzylamido)succinyl hydroxamic acid δ_H_ (400 MHz, CDCl_3_) 2.7 (2H, t, CH_2_CONOH), 2.95 (2H, t, CH_2_CONH), 3.82 (3H, s, OCH_3_), 4.28 (2H, s, CH_2_NH), 4.8 (2H, s, CH_2_NOH), 6.88 (2H, d, CHCHC(F)), 7.0 (2H, d, CHCHC(OMe)), 7.15(2H, d, CHC(F)), 7.20 (2H, d, CHC(OMe)). δ_C_ (175MHz, CDCl_3_) 30.0, 34.5, 43.8, 50.7, 55.0, 114.1, 115.5, 192.2, 130.5, 156.9, 159.1, 172.2, 173.0 ppm. HRMS obs. 383.1413. calc 383.1378 for C_19_H_21_N_2_O_4_FNa^+^. D14: *N*-(4-fluorobenzyl)-3,4-methoxycinnamic hydroxamic acid. δ_H_ (300MHz, CDCl_3_) 2.65 (2H, t, PhCH_2_), 2.90 (2H, t, CH_2_CO), 3.49 (3H, s, OCH_3_ (meta)), 3.65 (3H, s, OCH_3_ (para)), 4.64 (2H, s, NCH_2_), 6.75 (7H, m, C_6_H_3_ & C_6_H_4_).

### Tomato seed assays

Seed were obtained from tomato plants of Ailsa Craig, sp5 and sp12 (previously named “D9”). Different batches of seed were used throughout the study but the same batch was used within each experiment. Hydroxamic inhibitors (Sergeant et al., 2009) were dissolved at 0.5 M in DMSO to create stock solutions. These were then diluted to 1 mM in sterile distilled water for use in the germination assays. NaOH was added to a final concentration of 0.2 mM to increase D4 solubility. Controls contained 0.2% DMSO (unless otherwise stated) and 0.2 mM NaOH (for D4 control) in sterile distilled water and were shown not to affect germination rates compared to sterile distilled water. Sterile filter paper (8.5 cm diameter, Whatman No.1) was placed in a 9 cm triple vented Petri dish and 1.5 ml of appropriate inhibitor solution was added, except in **Figure 2** where the volume was reduced to 1 ml. Where it is stated that seeds were sterilized they were submersed in a 1% w/v solution of sodium hypochlorite for 20 min, and then rinsed in tap water for 2 min. Twenty or twenty-five wetted seed were evenly placed on the filter paper in each dish. Petri dishes were placed in sealed plastic boxes containing moist tissue paper, in the dark at 25°C. Germination was scored when the radicle protruded from the testa, and mean germination time was calculated as previously described (Rowse, 1996). For the reduced water potential experiments, a stock solution of polyethylene glycol (PEG), mean molecular weight 6,000 (Sigma, Poole, UK) at a concentration of 40% w/v in water was prepared and diluted with water to achieve the correct working concentrations. Petri dishes containing PEG solutions were sealed with Parafilm to prevent evaporation and maintain osmotic potential.

### ABA measurements

Ungerminated WT and sp12 seed imbibed in Petri dishes (described above) were removed at set intervals, washed twice in 200 ml of distilled water, blotted on tissue paper, frozen in liquid nitrogen, freeze dried and sent to the National Research Council Plant Biotechnology Institute in Saskatchewan, Canada for analysis of ABA and catabolites (Chiwocha et al., 2005).

### Lettuce seed assays

Seeds from two unspecified lettuce varieties were obtained from Nunhems, Netherlands. Seed were placed in 5 cm diameter Petri dishes containing 4.5 cm diameter filter paper (Whatman No. 1) wetted with 0.5 ml of D4 solution (1 mM D4, 0.2 mM NaOH, 0.2% DMSO in sterile distilled water) or control solution (0.2 mM NaOH, 0.2% DMSO in sterile distilled water). Twenty-five seed were placed in each Petri dish, incubated in a sealed box at 25° or 30°C and scored daily for germination (when the radicle first became visible).

### Tobacco seed assays

Tobacco seed containing *LeNCED1* driven by the tetracycline inducible promoter were as previously described (Thompson et al., 2000). Seed were sterilized with 10% v/v household bleach (Domestos, Unilever, UK) for 30 min, followed by five washes in sterile distilled water. Twenty-five seed were placed on Whatman No. 1 filter paper wetted with 1.5 ml of solution in a 9 cm triple vented Petri dish. Solutions contained the specified concentrations of tetracycline, D4, and (+)-ABA made by diluting the following stock solutions in sterile distilled water: 1 mg ml^−1^ tetracycline in ethanol; 0.5 M D4 in DMSO; 1000 × final concentration (+)-ABA in ethanol. Controls contained equivalent DMSO and ethanol concentrations to the treatments containing D4 and ABA. Seed were incubated at 25°C in the dark in sealed plastic boxes containing wetted tissues and scored daily for radicle and cotyledon emergence.

### Measuring effect of D4 on seedling vigor

Tomato seeds of line sp12 were placed on filter paper soaked in a solution with or without 1 mM D4, as described above. Over an interval of 7 days, on its day of germination each seed was transferred to Levington's F2 compost containing sand (Fisons, Ipswich, UK) and placed in a controlled environment cabinet set to a 16:8 h day:night light cycle with daytime irradiance of 400 μmol m^−2^ s^−1^, respective temperatures of 22 and 18°C, and a relative humidity of 80%. Each seedling was then harvested at either 5, 13, or 20 days after its individual germination date. For each treatment, 60 seed were germinated and height, fresh and dry mass of 20 seedling shoots was recorded after 5, 13, and 20 days of growth.

Seeds of tobacco harboring tetracycline inducible *LeNCED1* were sown on 0.6% agar containing ½ strength MS salts (Murashige and Skoog, 1962) in the presence of tetracycline concentrations of up to 0.2 mg l^−1^, 0 or 1 mM D4 and 0.2% DMSO in 25-well-plates. Seed were incubated at 25°C in the dark for 6 days before being transferred to a light:dark regime of 16:8 h each day. Plates were photographed on day 10 and ImageJ (Schneider et al., 2012) was used to quantify projected green leaf area.

## Results

### Screening for compounds that stimulate germination

**Figure 1:**
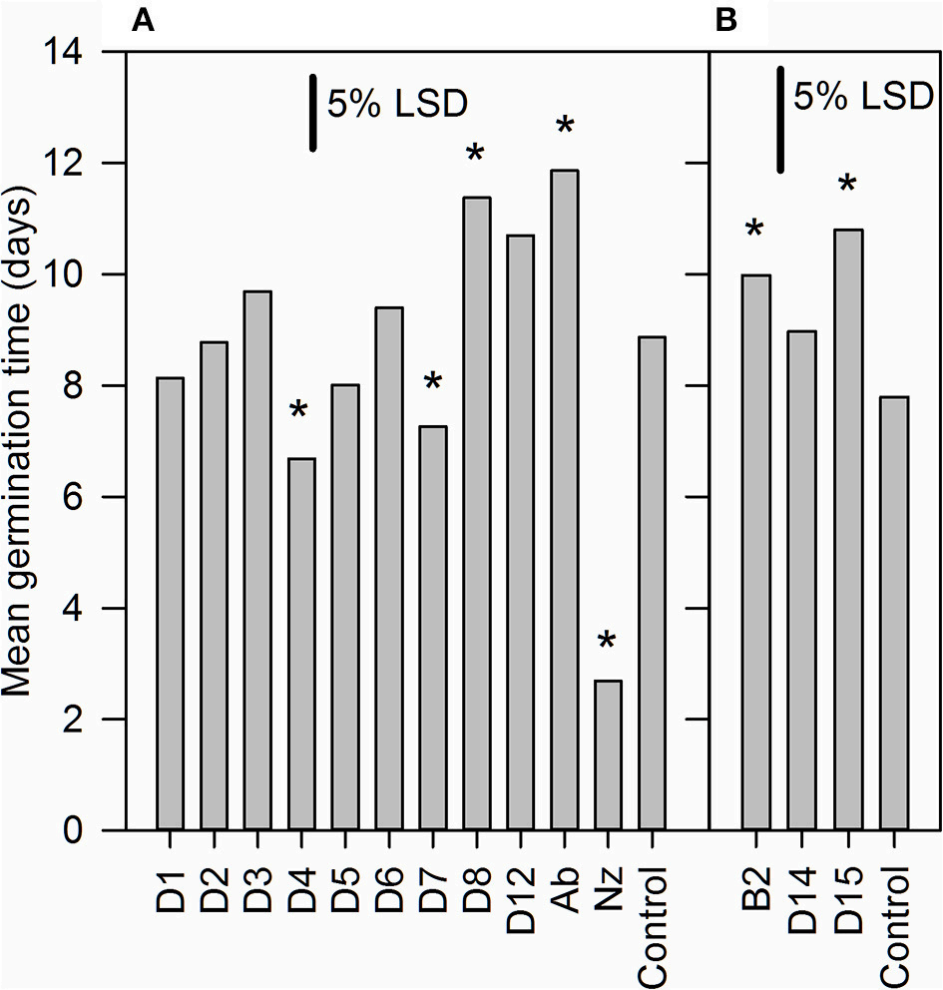
**Identification of hydroxamic acid compounds that promote seed germination**. Seed of an *LeNCED1*-overexpressing tomato line (sp12) were imbibed on filter paper wetted with 1 mM of 12 different hydroxamic acid compounds (D1–D8, D12, D14, D15, and B2), abamine (Ab), or 1.8 mg l^−1^ norflurazon (Nz). Each solution contained 0.2 mM NaOH and 0.2% DMSO, required for solubilization. The control solution contained only 0.2 mM NaOH and 0.2% DMSO. ^*^Indicates significant difference compared to control at *p* < 0.05. Two independent experiments were carried out in **(A,B)**, with the same control treatment. Floating bars represent least significant difference (LSD) between any two means within an experiment at 5% level, from ANOVA (*n* = 3).

To screen inhibitors for their ability to promote germination we used sp12 tomato seeds (Thompson et al., 2000, 2007) in which the over-expression of *LeNCED1* results in increased ABA biosynthesis and a 4–5 day delay in germination (Thompson et al., 2000). Twelve hydroxamic acids, some known to be CCD inhibitors (Table 1) and the previously identified NCED inhibitor abamine (Han et al., 2004) were screened for germination stimulating activity (Figure 1). Norflurazon was used as a positive control of germination stimulation. D4 and D7 significantly decreased the mean germination time (MGT) compared to control, whereas, B2, D8, D12, and abamine significantly increased mean germination time. Norflurazon decreased the mean germination time significantly more than D4 and D7.

**Figure 2:**
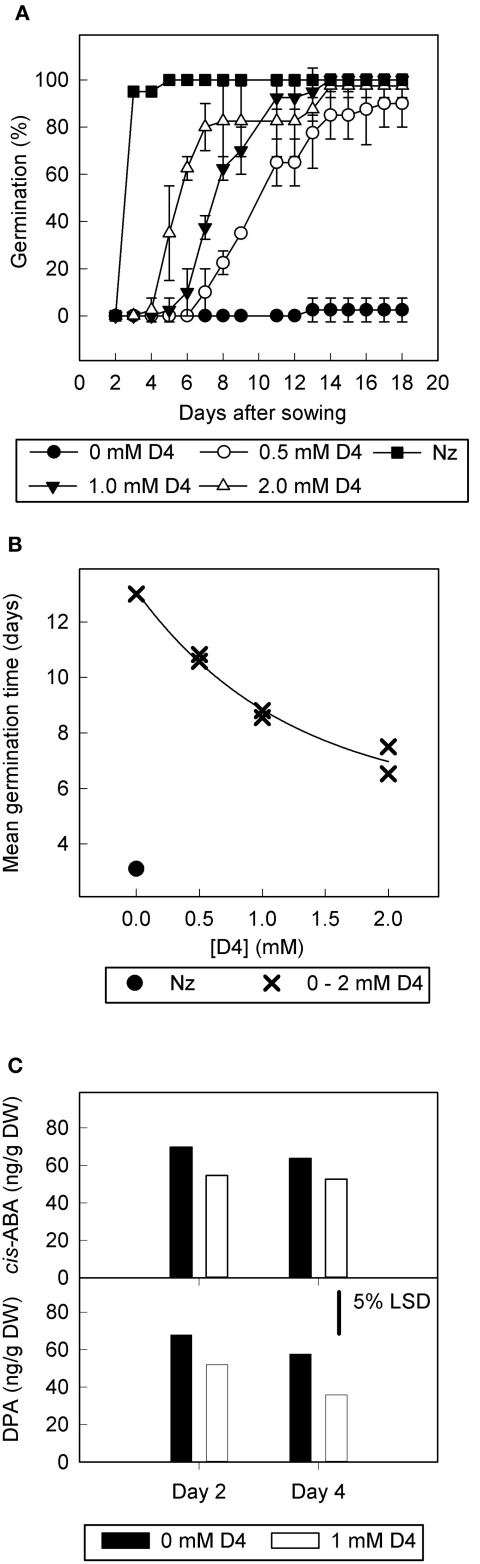
**The effect of D4 on germination, abscisic acid, and dihydrophaseic acid content of tomato seed overexpressing *LeNCED1***. Seed of transgenic line sp12 were imbibed on filter paper wetted with 0–2.0 mM D4, or with 1.8 mg l^−1^ norflurazon (Nz), in sterile water containing 1% DMSO and 0.2 mM NaOH. **(A)** Percentage germination over time plotted for 0 mM D4, 0.5 mM D4, 1.0 mM D4, 2.0 mM D4, and 1.8 mg l^−1^ norflurazon (Nz). **(B)** Mean germination time (radicle emergence) at 0–2.0 mM D4 and 1.8 mg l^−1^ norflurazon. **(C)**, abscisic acid (ABA) and dihydrophaseic acid (DPA) content of imbibed but ungerminated seed of sp12, imbibed with and without 1 mM D4 for 2 or 4 days. LSDs at the 5% level from ANOVA are indicated, *n* = 4.

D4 was tested further at different concentrations to determine dose-response (Figures 2A,B) in slightly different conditions to the original screen (1% DMSO rather than 0.2%, with seed sterilization and reduced ratio of compound solution to filter paper). These conditions inhibited germination more relative to Figure 1. In this case, the control treatment exhibited low final percentage germination (2.5%) and the addition of D4 allowed an increase in final percentage germination to ≥90% for all D4 concentrations (0.5, 1.0, and 2.0 mM; Figure 2A). Furthermore, D4 decreased MGT in a dose responsive manner, with the highest concentration of D4 (2 mM) reducing MGT by 5 days (Figure 2B). The positive control norflurazon was the most effective at reducing MGT and allowed 100% germination.

To determine the effect of D4 on ABA levels in the seed, the concentration of ABA and its derivatives were measured in sp12 seed prior to germination, at 2 and 4 days after imbibition. One millimolar D4 significantly decreased ABA and dihydrophaseic acid (DPA) concentrations by 20% (*p* = 0.002) and 30% (*p* = 0.024), respectively, when considering both time points together. There were no significant differences in ABA or DPA concentrations when comparing days 2 and 4, and no interaction between time point and addition of inhibitor. Concentrations of other ABA catabolites were too low to allow quantification.

### Chemical and genetic manipulation of ABA accumulation in tobacco seeds

**Figure 3:**
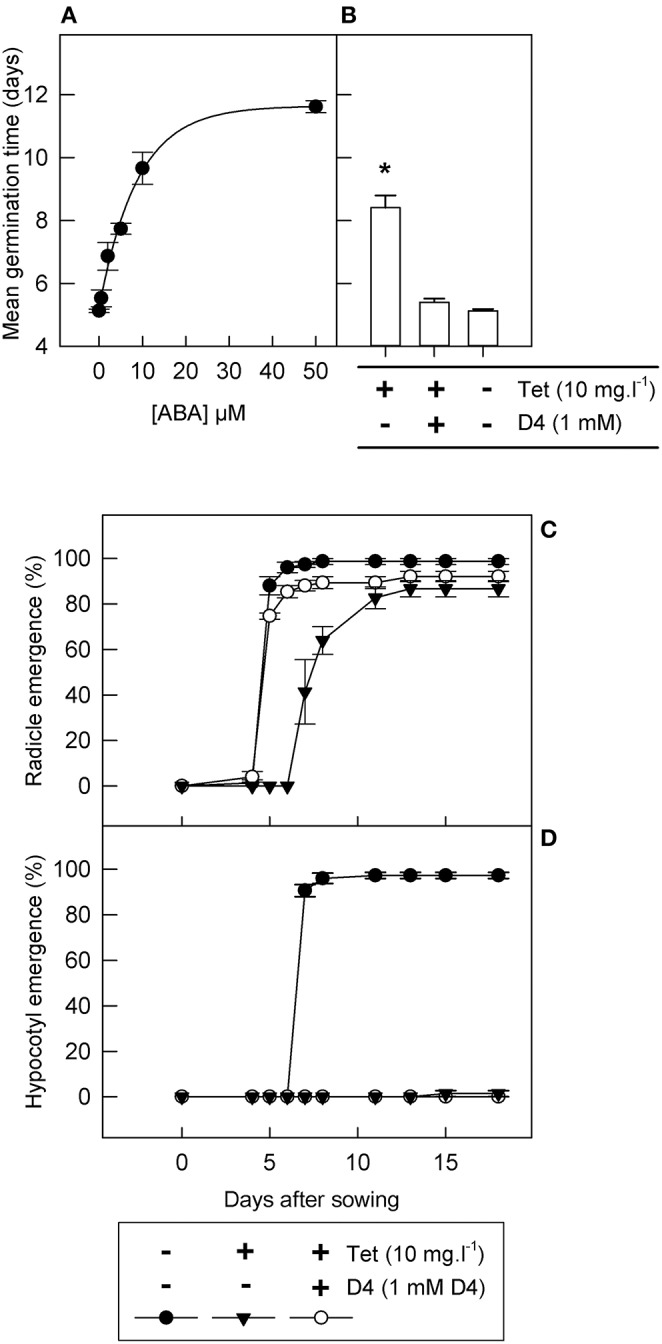
**D4 reverses the effect of inducible *LeNCED1* expression on radicle emergence in transgenic tobacco seed**. **(A)** Tobacco seed were imbibed in the absence of tetracycline on filter paper wetted with 0–50 μM ABA and mean germination time was calculated; the curve was fitted using a three parameter exponential rise to maximum model (*R*^2^ = 0.95; *p* < 0.001). **(B)** Tobacco seed were sown on filter paper wetted with or without 10 mg l^−1^ tetracycline and 1 mM D4, as indicated; all treatments additionally contained 0.2% DMSO, required to solubilize D4; ^*^indicates significant difference (*p* < 0.05) compared to 0.2% DMSO control, according to ANOVA least significant difference; in both **(A,B)**, mean germination time is based on radicle emergence. **(C,D)** show a time course of radicle emergence and hypocotyl emergence, respectively, with or without 10 mg l^−1^ tetracycline (Tet) and 1 mM D4, as indicated. Error bars indicate standard error (*n* = 3); all treatments also contained 0.2% DMSO.

Next, an inducible *LeNCED1* expression system was used to investigate the effectiveness of the hydroxamic acid D4 in inhibiting NCED *in vivo* and in promoting germination: this comprised transgenic tobacco seeds containing tetracycline inducible *LeNCED1* under the control of the Triple-Op promoter (Thompson et al., 2000). First, we established the response of these seed to *exogenously* applied ABA when *LeNCED1* was non-induced in the absence of tetracycline (Figure 3A). Radicle emergence occurred ~5 days after imbibition in the absence of both ABA and tetracycline, and increasing concentrations of ABA caused increasing delays in radicle emergence, with radicle emergence occurring later than 11 days at 50 μM ABA (Figure 3A). Hypocotyl emergence was totally prevented by ABA concentrations of 10 μM and above, and lower ABA concentrations partially inhibited emergence and led to abnormal hypocotyl phenotypes (data not shown).

To test the effect of *endogenously* synthesized ABA, seed were imbibed on solutions containing tetracycline to induce *LeNCED1* expression. Addition of 10 mg l^−1^ tetracycline resulted in a significant increase in mean radicle emergence time from 5.2 to 8.4 days, equivalent to imbibition on between 5 and 10 μM ABA (Figure 3B). Tetracycline concentrations above 0.5 mg l^−1^ indicated no clear dose-responsiveness because 10 mg l^−1^ tetracycline resulted in very similar effects to 0.5 mg l^−1^ tetracycline (data not shown). Addition of 1 mM D4 in combination with 10 mg l^−1^ tetracycline resulted in restoration of radicle emergence times, equivalent to seeds imbibed without tetracycline (Figure 3B). Imbibition on 10 mg l^−1^ tetracycline also reduced final percentage germination by 10%, which was partially restored by addition of 1 mM D4 (Figure 3C). Similar to treatment with 10 μM ABA, 10 mg l^−1^ tetracycline prevented hypocotyl emergence, but addition of 1 mM D4 was not able to reverse this effect (Figure 3D). In summary, tetracycline-induced *LeNCED1* expression results in a similar germination phenotype to that caused by exogenously applied ABA and D4 inhibits the effect of *LeNCED1* induction on radicle emergence.

### Effect of D4 on germination under sub-optimal and dormancy inducing conditions

**Figure 4:**
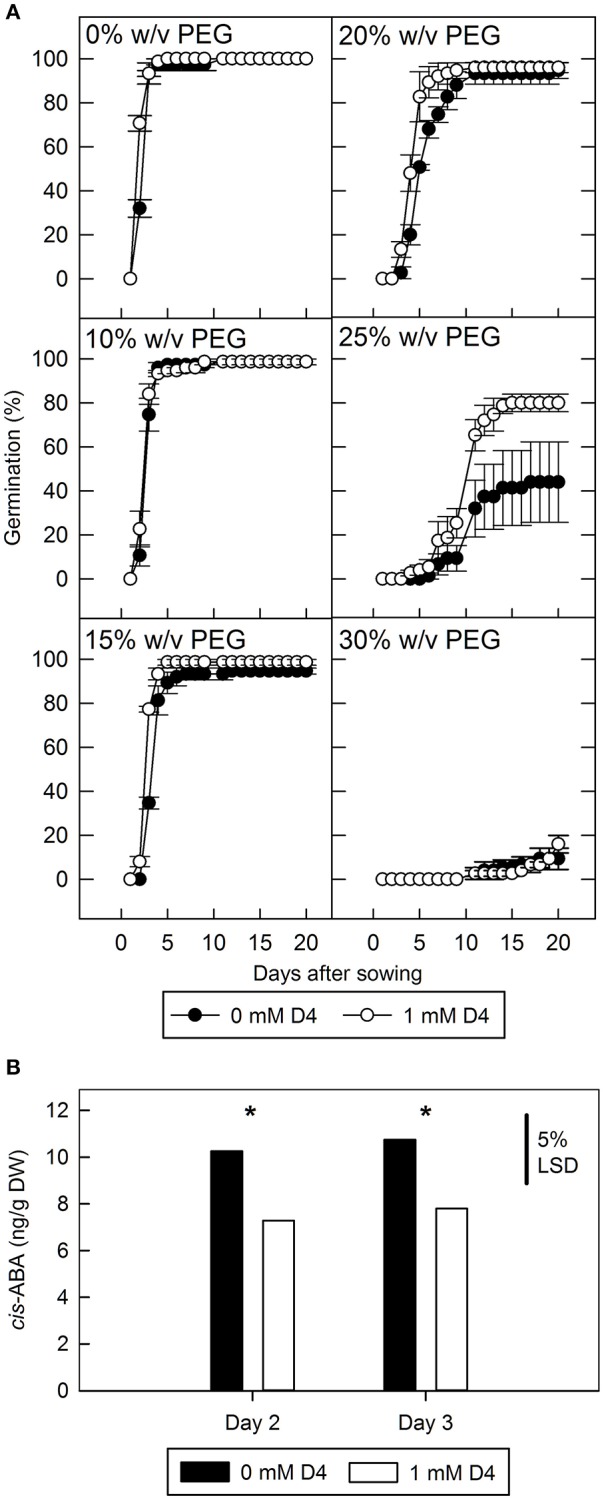
**The effect of polyethylene glycol (PEG) on tomato seed germination and abscisic acid (ABA) accumulation with and without D4**. **(A)** wild type tomato seed were imbibed on 0–30% w/v PEG with and without 1 mM D4, and radicle emergence was scored. Bars indicate standard error (*n* = 3). **(B)**, *cis*-abscisic acid was measured in ungerminated seed imbibed in 25% w/v PEG for 2 or 3 days with or without D4. LSD at the 5% level is indicated, and ^*^indicates a significant difference between 0 and 1 mM D4 treatments within each day (*p* < 0.05).

To show the effectiveness of D4 in improving germination in non-transgenic seed it was applied to seed imbibed in a range of polyethylene glycol (PEG; molecular weight = 6,000) concentrations (Figure 4A). At 10 and 15% w/v PEG there is little change in germination rate compared to water control, and consequently no effect of D4. At 30% w/v PEG only 9% germination occurred after 20 days and again D4 was observed to have no effect presumably due to the severity of the treatment. However, 25% w/v PEG allowed only 44% germination in the absence of D4 whereas the addition of 1 mM D4 stimulated germination to 80%, partially reversing the effect of PEG. At 20% w/v PEG, 1 mM D4 also increased the rate of germination, with the effect becoming most apparent between 5 and 7 days (Figure 4A). ABA and ABA catabolite content was measured in seed imbibed in 25% w/v PEG, but only ABA levels were sufficiently high to allow quantification: considering days 2 and 3 together, D4 reduced ABA accumulation by 28% (*p* < 0.05). However, it was noted that imbibition on 25% w/v PEG did not significantly increase ABA concentration compared to seed imbibed in absence of PEG after 2 days (data not shown).

**Figure 5:**
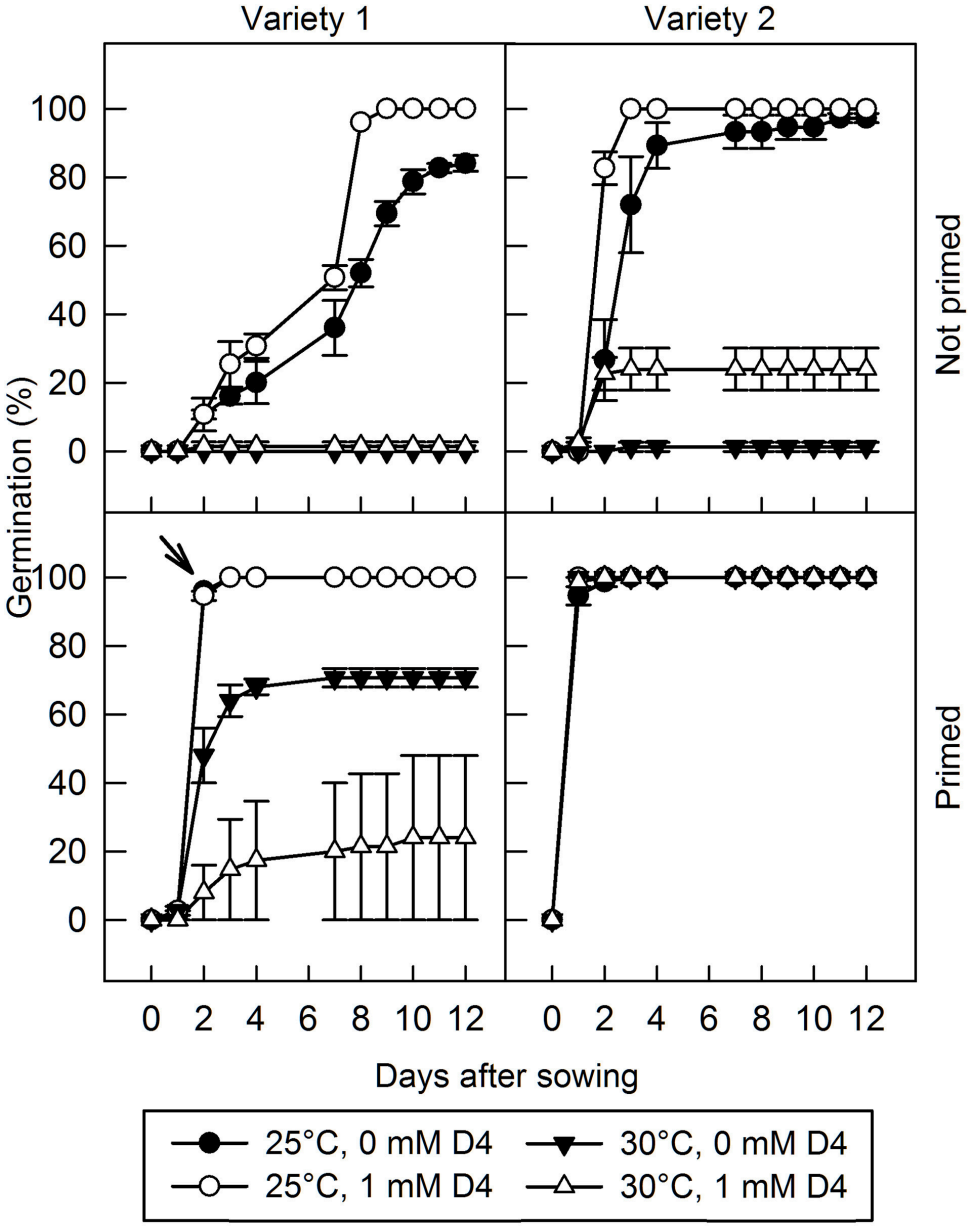
**The effect of D4 and priming on germination of two lettuce varieties at permissive and thermodormancy-inducing temperatures**. Lettuce seed of two different varieties were sown at 25°C and at 30°C with or without 1 mM D4, as indicated, and radicle emergence was scored. The arrow indicates where data for 0 mM D4 is obscured by the data for 1 mM D4 at 25°C. For the primed seeds of variety 2, much of the data overlaps due to rapid germination under all treatments. Bars indicate standard error (*n* = 3 plates each of 25 seeds).

Additionally, D4 was applied to two varieties of lettuce seed, imbibed under permissive (25°C) or thermoinhibitory (30°C) conditions, with or without seed priming (Figure 5). In variety 1, in the absence of D4 and priming, imbibition at 25°C allowed 84% germination after 12 days, whereas imbibition at 30°C completely prevented germination. Addition of 1 mM D4 improved germination slightly at 25°C, hastening germination and allowing 100% final germination, but there was no effect of D4 at 30°C. Variety 2 exhibited quicker and higher percentage germination than variety 1 at 25°C in the absence of priming, but like variety 1 was also almost completely thermoinhibited at 30°C; addition of D4 improved germination slightly at 25°C, but dramatically at 30°C from 1.3 to 24%. Seed priming is used commercially in lettuce to overcome thermoinhibition; germination of primed seeds was 100% for both varieties at 25°C whereas at 30°C final germination was 71 and 100% in varieties 1 and 2, respectively; priming therefore largely prevented thermoinhibition of germination. Interestingly, application of 1 mM D4 to primed seeds of variety 1 at 30°C inhibited germination, but otherwise D4 had no effect on germination of primed seed (Figure 5).

### Effect of D4 on seedling vigor

**Figure 6:**
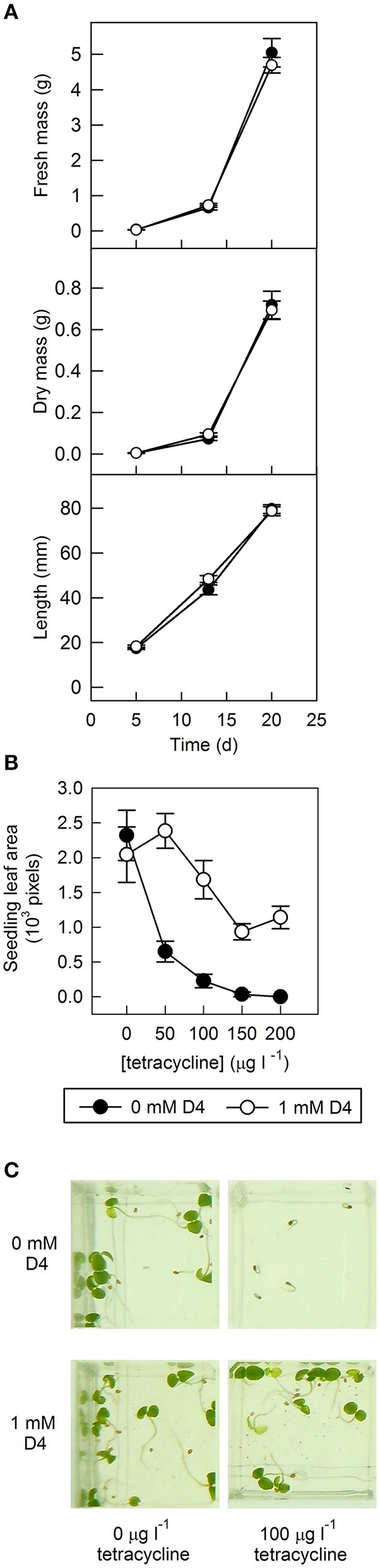
**The effect of D4 on seedling vigor in tomato and tobacco overexpressing *LeNCED1***. **(A)** *LeNCED1*-overexpressing tomato seed (sp12) were germinated on filter paper containing 0.1% DMSO with or without 1 mM D4, as indicated, and were transferred to soil following germination. Twenty shoots were harvested for measurement of dry mass, fresh mass, and length for each treatment at 5, 13, and 20 days post-germination. Error bars indicate standard error (*n* = 20). **(B)** Tobacco seed harboring tetracycline-inducible *LeNCED1* were sown on 0.6% agar containing ½ MS salts and 0–200 μg l^−1^ tetracycline with and without 1 mM D4 in 0.2% DMSO. Seed were incubated at 25°C in darkness for 6 days and then transferred to light conditions. Seedlings were photographed 10 days after sowing and green leaf area of seedlings was quantified using ImageJ. Error bars indicate standard error (*n* = 5). **(C)** Images of seedlings in plate wells representative of labeled treatments.

The effect of D4 and norflurazon treatment of tomato seeds on subsequent growth of seedlings was examined. As expected, norflurazon had a serious effect: seedling growth arrested when hypocotyls were ~10 mm long and was associated with a purple color, presumably due to accumulation of anthocyanins and photobleaching (data not shown). In contrast, D4 had no effect on seedling growth: length, fresh weight, and dry weight were similar to the control after 5, 13, and 20 days post-germination (Figure 6A).

In addition, tobacco seed harboring tetracycline-inducible *LeNCED1* were sown at a range of lower tetracycline concentrations than previously used (see Figure 3), with and without D4, and cotyledon area was measured as an indicator of seedling vigor (Figures 6B,C). Increasing concentrations of tetracycline led to reductions in cotyledon area after 10 days (Figure 6B), presumably due to *LeNCED1* induction and accumulation of ABA, although these low concentrations did allow radicle emergence (e.g., at 100 μg l^−1^, Figure 6C). In seedlings grown in the presence of 100 μg l^−1^ tetracycline, addition of 1 mM D4 restored cotyledon area, and visual phenotype to levels seen in seedlings germinated without tetracycline (Figure 6C). These results show that 1 mM D4 can reverse the inhibitory effect on seedling development that occurred upon induction of *LeNCED1*, and that 1 mM D4 had no effect on cotyledon area or visual seedling phenotype in the absence of tetracycline (Figures 6B,C), and so no non-specific inhibitory effect on seedling growth was observed.

## Discussion

### Discovery of the novel ability of hydroxamic acid NCED inhibitors to promote seed germination

We used sp12, a transgenic tomato seed line that exhibited delayed germination due to constitutive overexpression of *LeNCED1* and accumulation of ABA, to identify the two hydroxamic acid compounds D4 and D7 that were able to reverse seed dormancy (Figure 1), and subsequently demonstrated that D4 was able to deplete seed ABA levels (Figure 2). D4 and D7 were originally designed to inhibit the 9,10 cleavage activity of CCDs (Sergeant et al., 2009), based on the two carbon distance between the aryl group and the N of the hydroxamic acid (Aryl-C_2_N; Table 1). Although D4 and D7, did show moderate inhibition (33%) of LeNCED1 *in vitro* (Sergeant et al., 2009), they, along with other compounds in the D1-D13 series, all exhibited more effective *in vitro* inhibition of the 9,10/9′,10′ cleavage activity of LeCCD1a. Previously D8 showed the greatest specificity toward NCED because inhibition of LeCCD1a *in vitro* was 61% compared to >95% for the other hydroxamic acids, and inhibition of LeNCED1 was marginally the highest at 40% (Sergeant et al., 2009), however, D8 inhibited rather than promoted germination of sp12 seed. Likewise, abamine, which also inhibited LeCCD1a (35%) and LeNCED1 (20%) *in vitro* (Sergeant et al., 2009), inhibited germination of sp12 seed when applied at 1 mM; this was not surprising because although a positive effect was seen on Arabidopsis germination at 10–30 μM (Li et al., 2012), it was inhibitory to cress growth at 100 μM (Han et al., 2004). Sesquiterpene-like compounds designed to inhibit NCED also inhibited seed germination even though, like abamine, they do decrease ABA levels in osmotically stressed Arabidopsis plants (Han et al., 2004; Boyd et al., 2009). The overall lack of correlation between *in vitro* NCED inhibition and the ability to promote germination could be because inhibitors interact with unknown targets that inhibit growth and thereby mask the effects of ABA depletion, or they may fail to inhibit NCED *in vivo* due to a high rate of their catabolism, or poor uptake into the seed. Thus, apparently, D4 and D7 have properties in addition to their ability to act as enzymatic inhibitors that allow them to be effective in the promotion of germination. In part, this could be due to their lacking any toxic or general growth inhibitory effects even at 1 mM (Figure 6); as such they may have a unique utility in seed treatments as they are the only reported compounds able to deplete ABA content without non-specific growth inhibitory effects at concentrations of ≥100 μM. AbamineSG also has low toxicity to plant growth and development at 100 μM (Kitahata et al., 2006), but its ability to promote germination has not been reported.

### The chemical control of germination in tobacco seeds through inducible *LeNCED1* overexpression and NCED inhibition

The ability to chemically induce NCED gene expression, and to chemically inhibit the NCED enzyme, provides a fine chemical control of endogenous ABA accumulation in seeds. In order to put the inducible *LeNCED1* system in tobacco seeds in the context of the many studies of exogenously applied ABA, the dose effect of exogenous ABA application was calibrated against tetracycline-induced *LeNCED1* expression (Figures 3A,B). Seeds responded to tetracycline in the same way in the range 0.5–10 mg l^−1^ (data not shown), and the saturating concentration of 10 mg l^−1^ tetracycline provided a maximum increase in mean time to radicle emergence equivalent to 5 μM exogenously applied ABA.

Hypocotyl emergence was observed to be more sensitive to exogenous ABA than radicle emergence in tobacco seeds because 10 μM ABA gave a mean time to radicle emergence of 9.5 days (Figure 3A), but subsequent growth was completely arrested (data not shown). Similarly, *LeNCED1* gene expression induced by 10 mg l^−1^ tetracycline delayed radicle emergence, but also totally prevented hypocotyl emergence (Figures 3C,D). This is in agreement with a study in Arabidopsis seeds where methoxyfenozide-inducible *AtNCED6* expression led to ABA accumulation and growth arrest after endosperm rupture and radicle emergence (Martínez-Andújar et al., 2011). This supports the view that induced *NCED* mimics exogenously applied ABA by producing an arrested germination event rather than a dormant seed, and reinforces the idea that ABA biosynthesis is required but not always sufficient to induce true seed dormancy. The high sensitivity of the hypocotyl to ABA may be related to the post-germination growth-arrest checkpoint which is dependent on components of the ABA signal transduction pathway (Lopez-Molina et al., 2001, 2002).

When tetracycline was applied at the saturating concentration of 10 mg l^−1^, equivalent to 5 μM exogenous ABA, D4 reversed the effect of *LeNCED1* overexpression on mean germination time, but failed to restore hypocotyl emergence (Figure 3). However, when lower tetracycline concentrations (≤ 100 μg l^−1^) were used to induce *LeNCED1* expression, 1 mM D4 was able to restore hypocotyl emergence and a phenotype visually similar to seed germinating normally in the absence of tetracycline or D4. Therefore, D4 was only able to reverse the effects of a certain degree of *LeNCED1* overexpression, presumably because of its limited potency. Indeed, complete tobacco seed germination, including hypocotyl emergence and development of seedling leaf area was highly responsive to the tetracycline inducer in the range 0–100 μg l^−1^, and this effect was fully reversible by 1 mM D4 at 50 μg l^−1^ tetracycline and partially reversible between 100 and 200 μg l^−1^ tetracycline (Figure 6). Thus, by combining tetracycline and D4 chemical treatments, the full range of germination and seedling establishment stages can be dynamically controlled, from delayed radicle emergence, to growth arrest, to differential rate of cotyledon development.

### D4 promotes germination under osmotic stress in tomato and alleviates thermoinhibition of germination in lettuce

D4 was able to stimulate germination of wild type tomato seeds when imbibed on high osmoticum solutions of PEG. Although ABA levels were depleted by 25% in the presence of D4, the PEG treatment itself did not increase ABA content. The latter is consistent with previous findings that low water potential does not exert its effect by increasing the endogenous levels of ABA in the seed (Ni and Bradford, 1992). It is likely that the reduced levels of ABA caused by D4 application result in weakening of maternal tissues, allowing their rupture and germination of the seed at the lower rate of embryo expansion that occurs at low water potential.

Thermoinhibition of germination in lettuce is dependent on an increased production of ABA due to elevated expression of *LsNCED4* at higher temperatures (Argyris et al., 2008; Huo et al., 2013). It is therefore predicted that NCED inhibitors would reduce lettuce thermoinhibition. Indeed, D4 improved germination of lettuce under thermoinhibitory conditions in the absence of priming in variety 2 (Figure 5). Priming was more effective than D4 at reducing thermoinhibition, and in primed seeds of variety 1, D4 treatment *decreased* germination at 30°C; this was the only circumstance in which D4 had a negative impact on germination, and hypotheses about the inhibitory mode of action are given below.

### Mode of action of D4 and potential physiological consequences of inhibition of multiple CCD enzymes

It is reported that SLs enhance germination in Arabidopsis seeds at supra-optimal temperatures, a process that is dependent on modulation of ABA signaling (Toh et al., 2008), and involves a reduction in *AtNCED9* expression (Toh et al., 2012). Lettuce seed germination can also be improved by application of SLs (Bradow et al., 1988). Thus, there are two antagonistic mechanisms that both require CCD activity for their synthesis: ABA synthesis is dependent on NCED activity, and SL biosynthesis is dependent on CCD8 activity (Alder et al., 2012), both of which are inhibited by D4 *in vitro* (Sergeant et al., 2009; Harrison et al., 2015). However, retarded germination is not a reported phenotype of *ccd* mutants under optimal conditions, for example the Arabidopsis null mutants in genes *AtCCD7, AtCCD8*, or *AtCCD1* germinate as wild type (Auldridge et al., 2006). Therefore, under most seed germination environments, where SLs are not critical, D4 is predicted to enhance germination by inhibiting NCED. If both hormones are involved then D4 will have two antagonistic effects on germination, and if SLs dominate, D4 will inhibit germination. Because D4 increases germination in all circumstances with one exception, its normal mode of action is very likely to be to directly inhibit NCED, a conclusion supported by *in vitro* NCED inhibition, reversal of seed dormancy caused by over-expression of NCED in tomato and tobacco, and the observation that it reduces ABA content of transgenic and wild type tomato seeds.

The exception is in primed lettuce seeds (variety 1; Figure 5) germinated at high temperature (30°C) where D4 inhibited germination. In this case it is hypothesized that priming negates the normal role of NCED because germination has been initiated and arrested by high osmoticum (Taylor et al., 1998), but that SL retains a positive role in promoting germination at high temperature. In this situation, D4 can only act through reducing SL biosynthesis, which would tend to slow germination. In contrast, in the absence of priming, NCED retains its regulatory role and is proposed to be the dominant hormone-related CCD in lettuce thermoinhibition (Argyris et al., 2008; Huo et al., 2013), hence the D4-mediated increase in germination in unprimed variety 2 at 30°C (Figure 5). It may be possible in future studies to determine the target of D4 under these situations by quantifying both ABA and SLs or using novel inhibitors that are specific to individual CCDs or NCED. However, such studies may also have to consider the cross-talk that may occur between ABA and SL signaling pathways (López-Ráez et al., 2010).

### Impact of moderate changes in seed ABA content

D4 caused a drop in seed ABA content of 20 and 25% in transgenic (Figure 2) and physiological experiments (Figure 4), respectively, and this drop was associated with increased germination. Although a sharp drop in ABA is observed in seed induced to germinate with norflurazon, the observation that a moderately reduced level of ABA can increase germination is consistent with the ABA-time model (Ni and Bradford, 1992). In this model, there is a threshold ABA level, above which a seed will not germinate. ABA-time is calculated as the cumulative difference between this threshold and the actual ABA level in the seed multiplied by time; it more accurately predicts seed behavior than a simple threshold model. The experiments presented here appear to support this model, because it predicts that even a small decrease in ABA levels maintained over a time period can significantly decrease mean germination time, as was observed with application of D4.

Moderate inhibition of NCED by D4 may have reduced the ABA levels below this threshold in some of the lettuce seeds of variety 2 imbibed at 30°C, but not in variety 1 imbibed at 30°C (Figure 5). Thus, it is possible that lettuce seed of variety 1 have a lower ABA threshold level, or accumulate more ABA, compared to seed of variety 2. Indeed, variety 1 also appears to have increased dormancy relative to variety 2 even at 25°C which can only be partially alleviated by application of D4. Studies of ABA accumulation and sensitivity, or studies using novel NCED inhibitors with increased potency may yield further insight into the role of NCED in lettuce germination inhibition under these conditions.

## Conclusions

Transgenic seed lines over-expressing NCED have been used to discover novel NCED inhibitors, including compound D4, which improve seed germination in situations where increased ABA levels are the cause of reduced rates of germination such as in thermoinhibition of lettuce seed or when ABA levels are increased due to overexpression of NCED. In addition, this study implies that even when germination inhibition is caused by other factors apart from increased ABA, a reduction in the basal ABA level can still lead to improved germination. This was the case with tomato seeds grown in high osmoticum, where ABA levels did not increase, but reduction of ABA levels by D4 was still able to compensate for the factors delaying germination. This study has also shown that lowering the ABA content of the seed only slightly can still have a significant effect, since even a small decrease in ABA over a prolonged period could enhance germination, which would be predicted by the ABA-time model. The ability to dynamically control endogenous ABA synthesis in seeds or other tissues with a combination of chemical induction of NCED, and inhibition of NCED provides a new approach to dissect functional roles of ABA.

D4 is an attractive lead chemical with which to manipulate germination because it is the only NCED inhibitor known to enhance germination under a range of conditions without negative impacts on seedling growth at concentrations as high as 1 mM. Further, work is required to improve the potency and specificity of compounds based on the D4 chemical structure.

## Author contributions

AT and TB conceived the project. TB designed the hydroxamic acid inhibitors. AT developed and provided the transgenic tomato and tobacco lines with modified NCED expression and designed the seed germination assays. SA, JC, and MS performed the physiological experiments. PH performed chemical synthesis. JC and SA analyzed the data. JC and AT wrote the manuscript with contributions from all authors.

## Funding

The work was supported by BBSRC (grant BB/D005787/1 awarded to AT and TB and supporting MS). JC was supported by a studentship funded by Syngenta Limited. PH was supported by a BBSRC CASE studentship, part funded by Syngenta. Data underlying this study can be accessed through the Cranfield University repository at http://dx.doi.org/10.17862/cranfield.rd.4246577.

### Conflict of interest statement

AT and TB are inventors on patent “Plant development control composition,” patent number: 9210928, which relates to work described in this manuscript. The other authors declare that the research was conducted in the absence of any commercial or financial relationships that could be construed as a potential conflict of interest.
